# Influence of Visual Prism Adaptation on Auditory Space Representation

**DOI:** 10.1177/2041669517746701

**Published:** 2017-12-18

**Authors:** Klaudia Pochopien, Manfred Fahle

**Affiliations:** Department of Human-Neurobiology, University of Bremen, Bremen, Germany

**Keywords:** prism adaptation, directional hearing, sound localization, perceptual learning, plasticity

## Abstract

Prisms shifting the visual input sideways produce a mismatch between the visual versus felt position of one’s hand. Prism adaptation eliminates this mismatch, realigning hand proprioception with visual input. Whether this realignment concerns exclusively the visuo-(hand)motor system or it generalizes to acoustic inputs is controversial. We here show that there is indeed a slight influence of visual adaptation on the perceived direction of acoustic sources. However, this shift in perceived auditory direction can be fully explained by a subconscious head rotation during prism exposure and by changes in arm proprioception. Hence, prism adaptation does only indirectly generalize to auditory space perception.

## Introduction

Interactions with the environment, such as grasping a remote, opening a window, or playing tennis (Baraduc & Wolpert, 2002; Kitazawa, Kohno, & Uka, 1995), rely on highly complex sensory-motor coordination (Crawford, Medendorp, & Marotta, 2004). A goal-directed movement requires a motor command based on both visual and acoustic information about target position and proprioceptive information about the position of the limb (Baraduc & Wolpert, 2002; Crawford et al., 2004). One remarkable function of this system is its ability to quasi automatically adapt to changes, for example, while wearing ordinary or prism glasses. After adaptation, the system functions well under the new conditions (Baraduc & Wolpert, 2002; Guan & Wade, 2000; Redding & Wallace, 1988; Rossetti, Koga, & Mano, 1993).

Adaptation to prism glasses is an excellent example for modifying the eye-hand coordination system (Rossetti et al., 1993). Prism glasses shift the visual (while not the acoustic) environment in the direction of the prismatic shift, causing errors when people reach toward visual objects (Bedford, 1995; Efstathiou, 1969; Guan & Wade, 2000; Harris, 1963, 1965; Hay & Pick, 1966; Redding, Rossetti, & Wallace, 2005; Redding & Wallace, 2006; Taub & Goldberg, 1973). This discrepancy between the visual input and hand proprioception is perceived and induces prism adaptation, as is the case in other adaptations to altered sensory environments (Welch, 2013; Welch & Cohen, 1991; Zwiers, van Opstal, & Paige, 2003).

The initial error of directed movements is termed the *direct effect* (Redding & Wallace, 1993; Redding et al., 2005). Through practice, the error rapidly diminishes and (almost) disappears due to prism adaptation (Baraduc & Wolpert, 2002; Bedford, 1995; Cohen, 1967; Efstathiou, 1969; Guan & Wade, 2000; Harris, 1963, 1965; Hay & Pick, 1966; Redding et al., 2005; Redding & Wallace, 2006; Taub & Goldberg, 1973). After removing the prism glasses, the adaptation decreases exponentially and a grasping error opposite to the prismatic displacement appears, the so-called *aftereffect* which largely disappears after less than 10 movements (Bedford, 1995; Cohen, 1967; Guan & Wade, 2000; Redding et al., 2005; Taub & Goldberg, 1973).

Since the end of the 19th century, many studies investigated prism adaptation (Efstathiou, 1969; Harris, 1965; Hay & Pick, 1966; Helmholtz, 1867) but many open questions remain. One of these open questions is whether directional hearing is affected by prism adaptation. In everyday life, the visual and acoustic representations coincide rather precisely and influence each other. We hear sound to come from objects we know to produce sounds, and may modify the perceived direction of sound according to visual input, as in the ventriloquist effect (Callan, Callan, & Ando, 2015; Chen & Spence, 2017) and aftereffect (Bosen, Fleming, Allen, O’Neill, & Paige, 2017; Radeau & Bertelson, 1974).

The present study investigates the influence of horizontally shifting prism glasses on acoustic localization. When an acoustic source is not exactly in front of the head (or behind it), sounds coming from this source reach the ears at different times. The ear closer to the acoustic source will be stimulated first. This results in a time-of-arrival difference between both ears, termed *interaural time difference*. A second source of sound localization is the interaural loudness level difference. The head produces an acoustic shadow. A sound coming from a lateral position achieves a higher sound pressure level in the nearer ear than in the one farther away (Blauert, 1983; Hartmann, Rakerd, Crawford, & Zhang, 2016; Hofman & van Opstal, 2003; Lewald, 2012; Macpherson & Middlebrooks, 2002; Middlebrooks & Green, 1991; Rayleigh, 1907; Schnupp, Nelken, & King, 2011).

In the past, a number of studies described that indeed adaptation to prisms, that is, to a change in visual space perception, influences the perception of acoustic space (Bosen et al., 2017; Callan et al., 2015; Chen & Spence, 2017; Radeau & Bertelson, 1974). On the other hand, at least three studies failed to find such an influence (Harris, 1963; Hein & Held, 1960; Pick, Hay, & Pabst, 1963).

Investigating the effects of prism adaptation, we tested directional hearing both before and after prism adaptation by having participants (a) pointing toward acoustic signals and (b) indicating sound direction relative to body straight ahead that is without influence of hand proprioception. We do find an effect of prism adaptation on acoustic localization, but this shift is not genuinely of acoustic nature.

## Material and Methods

### Ethics Statement

This study was approved by the local ethics committee of the University of Bremen. Prior to the study, participants were informed about the aim and procedure and had to sign a written consent form according to the Declaration of Helsinki (2008). Participants were paid for participation and were free to withdraw from the study at any time.

### Participants

Thirty-eight participants, aged 19 to 30 years, participated in three experiments. All were students of the University of Bremen. Participation criteria were right-handedness, normal or corrected-to-normal visual acuity (correction by contact lenses only; Freiburger Visual Acuity Test; Bach, 1996), normal stereopsis (Lang Stereo Test; Lang, 1983), no prior participation in prism experiments, and a pupillary distance between 54 and 64 mm for the right-shifting prisms and 59 and 69 mm for the left-shifting prisms (Auto-Refractometer, NIDEK ARK-700). Participants also had to be sufficiently capable to localize sounds by means of a two alternative forced choice by indicating whether a broadband tone of around 40 to 50 db duration and a sound level pressure of 320 ms produced by a computer via soundcard came from the left or else from the right. In addition, eye dominance was tested.

Experiment 1 comprised one group of 20 participants (9 females, 11 males; *M* = 25.1 years, *SE* = 0.68). In Experiment 2, seven additional participants (5 females, 2 males; *M* = 23.3, *SE* = 1.16) participated and in Experiment 3, an additional group of 11 participants (3 females, 8 males; *M* = 21.2, *SE* = 0.33) took part.

### Experiment 1: Auditory Localization During Adaptation in the Dark

#### Experimental set-up

**Figure 1.**
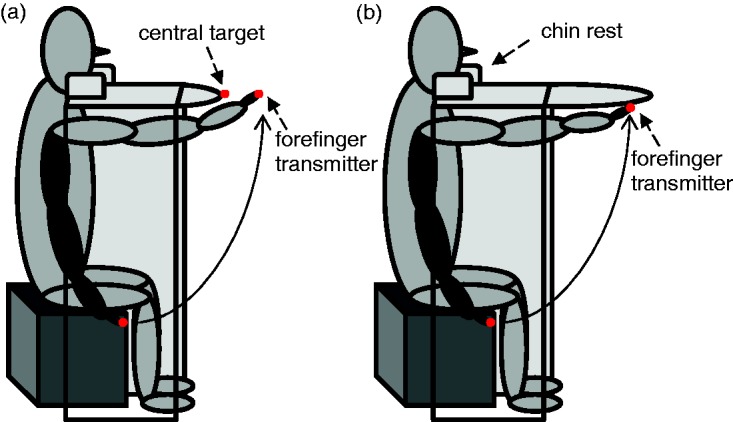
Schematic illustration of a participant executing a pointing movement toward (a) the central target with terminal feedback and (b) straight ahead without feedback. The black arm illustrates the starting position, the gray arm the movement endpoint. The red dots depict the central target as well as the finger transmitter.

Participants were seated at a table (99.5 cm high, 110 cm wide, and 57 cm deep [shorted table] or 76 cm deep [extended table]). A red diode was mounted centrally at the front side of the tabletop. The opaque tabletop occluded arm movements, blocking feedback on hand position either completely (extended table; Figure 1(b)) or incompletely (terminal feedback; Figure 1(a)) where the anterior 4 cm of the forefinger were visible at the movement’s endpoint. Participants performed arm movements with the right arm underneath the tabletop. A second diode was attached at the tip of the forefinger. A miniature piezo-electric ultrasound transmitter allowed tracking the trajectory of this finger in three dimensions. A chin rest kept the head-target distance at 51 cm.

**Figure 2.**
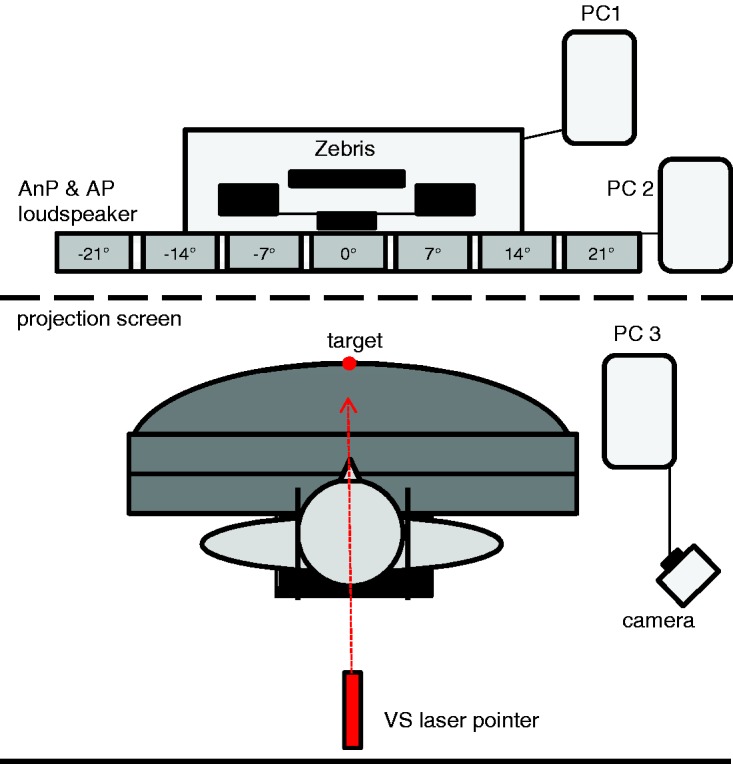
Schematic illustration of the test set-up. The participant sits at the table–chair. The red dot illustrates the position of the central target (0°). A white projection screen (black dashed line) positioned in front of the participant receives beams of the visual straight ahead laser pointer (VS; red). Seven loudspeaker spaced at 7° intervals (from −21° to 21°) behind the projection screen (invisible for the participant) emitted broad band sounds (gray rectangles) for measuring the nonproprioceptive auditory straight ahead (AnP) and proprioceptive auditory straight ahead (AP).

A movable laser pointer mounted on a tripod, localized behind the participant, produced a light spot on a projection screen located at 120 cm in front of the participant to measure subjective straight ahead (VS). A digital camera recorded positions of the laser beam (remote capture). Behind the projection screen (invisible for the participant), seven loudspeakers which could emit a broadband signal of around 40 to 50 db sound level pressure were placed at constant intervals of 7° (from 21° left to 21° right) at 88 cm height and a distance of 170 cm from the participants. An ultrasound measuring system (Zebris medical GmbH) tracked the movements of the forefinger transmitter and of the target transmitter (Figure 2). During the experiment, the room was absolutely dark.

The right-shifting prisms (Carl Zeiss, Oberkochen/Germany) had an optical center distance of 59 mm, with a prismatic effect of 25.4 prism diopters (cm/m), corresponding to a visual shift of 14.2°. The left-shifting prisms had an optical center distance of 64 mm with a prismatic shift of 25.8 prism diopters (cm/m) inducing a visual shift of 14.5°.

#### Procedure

**Figure 3.**
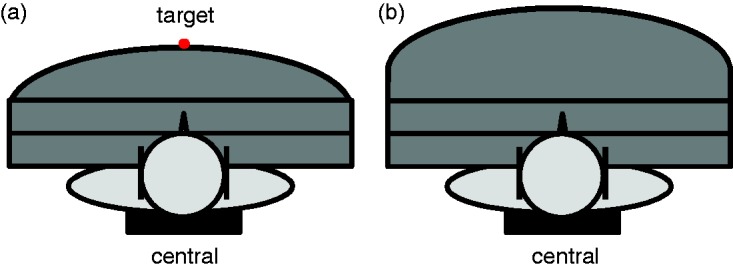
Schematic illustration of the test set-up: (a) for measuring adaptation and readaptation; with terminal feedback and (b) for measuring the nonproprioceptive auditory straight ahead (AnP), visual straight ahead, proprioceptive straight ahead (PS), and proprioceptive auditory straight ahead (AP) without feedback. The red dot indicates the central target (0°).

The participants of Experiment 1 performed rhythmic pointing movements toward a central target with terminal feedback (via finger-tip diode) to test baseline, adaptation, and readaptation with room lights off (Figure 3(a)). Each participant was tested twice, using both prism types (right-shifting and left-shifting) in counterbalanced order. Before adaptation, pretests were performed, followed by the first and second posttest after prism adaptation and readaptation, respectively, in the dark room and without feedback. These measurements determined the subjective visual straight ahead (measuring the straight ahead position of the eyes without visual feedback—VS), the subjective proprioceptive straight ahead (pointing in the midsagittal body direction without visual feedback—PS), and two types of subjective auditory straight aheads (evaluating the direction of the unseen auditory source without visual feedback and pointing movements—AnP, pointing toward an unseen auditory source without visual feedback—AP see below; Figure 3(b)).

Starting with the baseline measurement, the participant was asked to keep the head in the chin rest, to open the eyes, and to perform 30 rhythmic pointing movements with terminal feedback at a frequency of around 0.37 Hz (∼2.7 s per movement) toward the central target without prism glasses.

Next, we measured the *subjective nonproprioceptive AnP* by means of a two alternative forced choice (eyes closed) with participants indicating whether a broadband tone produced by a computer via soundcard came from the left or else from the right (all seven loudspeakers were used in turn; altogether 70 sounds (10 per loudspeaker)). The results were plotted as percentages correct for each loudspeaker position and an inverted Gaussian was fitted to these results, with both the outmost right and left loudspeakers usually yielding 100% correct responses. The minimum of this fit (50% point of a cumulative Gaussian) was considered the point of subjective equality and its deviation from the midsagittal plane is the effect of the prisms on auditory localization. The head was in the chin rest.

*Subjective VS* tests a possible shift in the spatial map for the visual domain. The participant aimed to look straight ahead while the laser beam moved slowly horizontally from right to left or vice versa on the projection screen. Without wearing prisms, the participant said when the spot on the screen appeared to be located exactly straight ahead, that is, in the center of their visual field. Each trial consisted of five movements, each documented by a digital photo.

To test *subjective PS*, participants closed their eyes (no visual feedback) and performed five slow pointing movements to the subjective straight ahead position, that is, along the body’s midsagittal plane.

To measure the *subjective proprioceptive AP*, broadband noise originated pseudorandomly from one of the seven loudspeakers. Participants pointed toward where they perceived the sound. Each data point relies on 35 presentations, distributed equally over the loudspeakers. The mean distance between pointing direction and actual position of the corresponding loudspeaker is one way we measured the mean shift of auditory localization from the midsagittal plane.

During the adaptation, participants wore prism glasses while performing 30 pointing movements with terminal feedback. Thereafter, participants performed the first posttest which is performed similar to the pretest and followed by another 30 pointing movements toward the central target with terminal feedback, without wearing prism glasses during readaptation. A second posttest again similar to the pretest followed after readaptation (see Table S1).

### Experiment 2: Auditory Localization During Adaptation in the Light

#### Experimental set-up and procedure

The only difference to the first experiment was that the lights were on during baseline measurement, adaptation, and readaptation. The prism spectacles had optical side shields. Pretest and posttests were measured in the dark (Table S9). Earlier, we found that the direct prism effect is smaller while the change in subconscious head rotation is larger in the light condition, and hence, the influence of head rotation on acoustic localization should be larger in the light.

### Experiment 3: Auditory Localization During Adaptation in the Light

#### Experimental set-up

**Figure 4.**
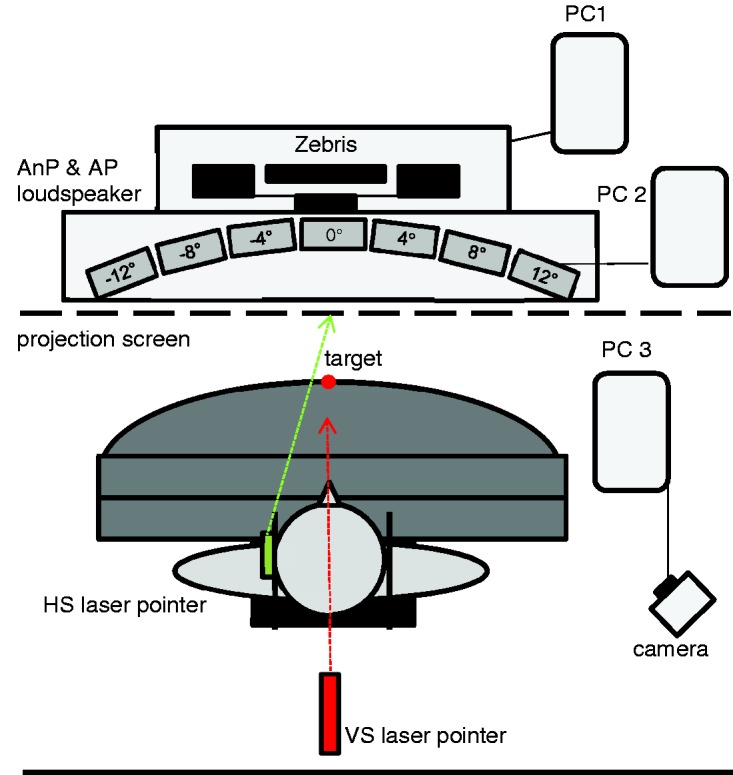
Schematic illustration of the test set-up. Participants sat at the table without head fixation. The red dot illustrates the position of the central target (0°). A white projection screen (black dashed line) positioned in front of the participant received beams of the movable visual straight ahead laser pointer (VS; red) as well as of the head laser pointer (HS; green). Laser positions were recorded by the digital camera mounted behind the participant. Seven loudspeaker spaced at 4° intervals (from −12° to 12°) behind the projection screen (invisible for the participant) emitted broad band sounds (gray rectangles) for measuring the nonproprioceptive auditory straight ahead (AnP) and proprioceptive auditory straight ahead (AP).

Experiment 3 was tested with room lights on and loudspeakers placed at intervals of 4° between −12° left and 12° right. Participants wore a forehead strap with a switchable laser pointer oriented toward the screen to measure the subjective head straight ahead (HS; Figure 4).

#### Procedure

Procedure differed slightly from Experiment 2 in that adaptation required 60 rather than 30 arm movements and the adaptive components were tested only during pretest and first posttest. The longer adaptation period, together with a larger head rotation, might have an influence on the amount of (acoustic) adaptation. Participants indicated (a) subjective AP, (b) subjective AnP, (c) subjective head straight ahead (HS), (d) subjective PS, and (e) subjective VS. The pretests were performed without prisms, while during the first posttest, the prisms were kept on except for the subjective VS.

In an additional test, subjective head straight ahead was measured five times with the eyes closed and participants aligned their head so that they perceived it as straight on their shoulders (documented by a digital photo; Table S18).

### Analysis

The ultrasound measuring system Zebris (Zebris Medical, Isny/Germany) recorded the participant’s arm trajectories in three dimensions (*x*-, *y*-, and *z*-axis). A MATLAB program (R2013a) developed in-house then identified the vertical extremum of each pointing movement. The position of the laser pointer spot was analyzed by means of another MATLAB program (R2013a) developed in-house which calculated the correct position on the projection screen by removing errors caused by parallax between the positions of participants versus camera. All results supplied by the programs were verified visually on the original data by the experimenter. We compared the results using analysis of variance (ANOVA) and *t* tests.

## Results

### Experiment 1: Auditory Localization During Adaptation in the Dark

The effect of prisms is more pronounced in the dark than in a bright room due to a smaller immediate correction effect in the dark (see Pochopien & Fahle, 2015). We chose this procedure in order to produce a better visual aftereffect that would allow better clarity whether or not a transfer occurs from visual to acoustic adaptation. For each participant, an individual baseline (baseline and pretest results for each adaptive component) was subtracted from the corresponding measurements after adaptation, readaptation, and first posttest.

#### Direct effect and aftereffect in the dark

A two-way ANOVA for repeated measurements was performed first with prism direction (right, left) and test (direct effect, aftereffect) as within-subject factors and illumination (Experiment 1: dark, Experiment 2: light) as between-subject factor. For statistical analysis, the results for left-shifting prisms were inverted. The main effect of prism direction, *F*(1, 25) = 0.0, *p* = .989, indicated no significant difference. Therefore, the results of both prism orientations were combined.

After calculating a one-way ANOVA for repeated measurements with test (direct effect, aftereffect) as within-subject factor and illumination (Experiment 1: dark, Experiment 2: light) as between-subject factor, the main effect test, *F*(1, 52) = 334.9, *p* ≤ .001, indicated a significant difference between direct effect and aftereffect. For verification, a two-sided *t* test for paired samples as post hoc analysis was calculated. Hence, a significant difference between the direct effect and the aftereffect (*t* = 16.5, *p* ≤ .001) exists (Table S2).

**Figure 5.**
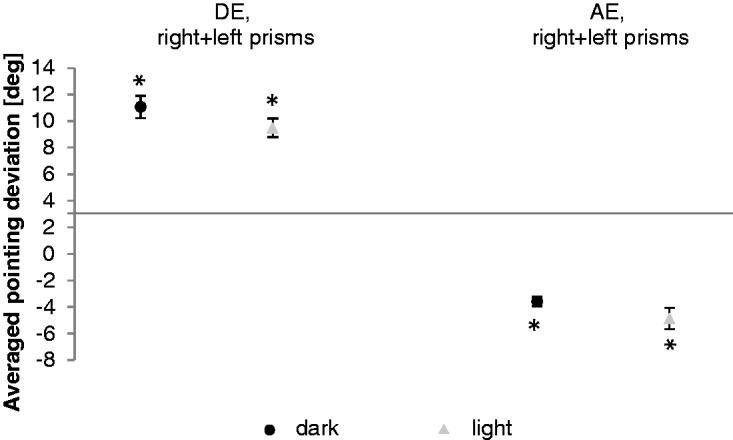
Direct effects (DE) and aftereffects (AE). Mean results of the right- and left-shifting prisms. Black dots show the averaged direct effects and aftereffects in the dark. Gray triangles show the averaged direct effects and aftereffects in light conditions. Error bars denote SEs. Asterisks indicate significant differences (*p* < .05).

Figure 5 shows the averaged and baseline-corrected direct effect (first pointing movement). A significant initial pointing error emerged (*M* = 11.1°, *SE* = 0.8, *t* = 13.4, *p* ≤ .001 one-sided *t* test against zero). The direct effect in darkness amounted to 78% of the prism’s optical power (Tables S3 and S4).

During readaptation, the first pointing movements deviate significantly in the direction opposite to the prismatic shift (*M* = −3.6°, *SE* = 0.3, *t* = −10.4, *p* ≤ .001 one-sided *t* test against zero), deviating by only 25% of prismatic power (Tables S3 and S4).

#### Adaptive components in the dark

A two-way ANOVA for repeated measurements was computed with prism direction (right, left) and posttest (VS, PS, AnP, and AP) as within-subject factors and illumination (Experiment 1: dark, Experiment 2: light) as between-subject factor. Please note that for statistical analysis, results for left-shifting prisms were inverted. The main effect of prism direction, *F*(1, 25) = 0.9, *p* = .343, was not significant. Therefore, we decided to combine the results of both prism orientations (mean of right and left prisms).

The one-way ANOVA for repeated measurements with posttest (VS, PS, AnP, and AP) as within-subject factor shows that the main effect of posttest, *F*(3, 156) = 9.9, *p* ≤ .001, has a significant influence on the different components of adaptation. For verification, two-sided *t* tests for paired samples as post hoc analysis were calculated. The subjective AP did not differ significantly from the subjective PS (Table S5). The results of the subjective AnP differed significantly from subjective VS (*t* = −2.7; *p* = .009; Table S6).

**Figure 6.**
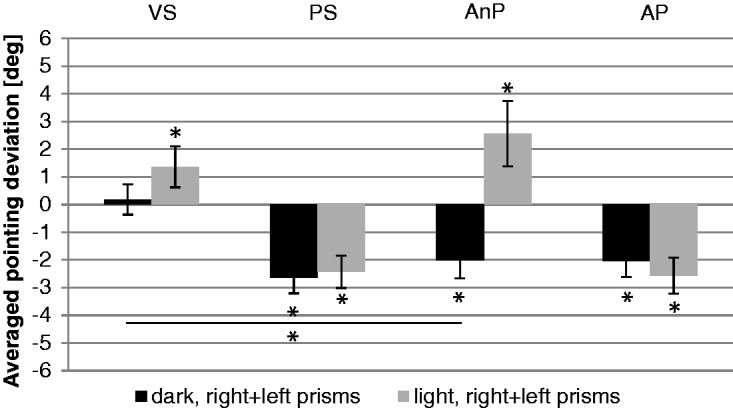
Adaptive components after prism adaptation (first posttest). Mean results of the right- and left-shifting prisms. Dark bars: dark condition (Experiment 1); light bars: light condition (Experiment 2). Bars indicate the averaged group results for the adaptive components (visual straight ahead [VS], proprioceptive straight ahead [PS], nonproprioceptive auditory straight ahead [AnP], and proprioceptive auditory straight ahead [AP]). Error bars indicate SEs. Asterisks indicate significant differences (*p* < .05), *T*s indicate trends (*p* < .1); asterisks with bars indicate significant differences (*p* < .05) between conditions, and *T*s with bars indicate trends (*p* < .1) between conditions.

Figure 6 shows the averaged and baseline-corrected results for subjective eye position (VS) and arm straight ahead position (PS) as well as two measures of the subjective auditory shift (AnP and AP). Subjective VS showed no significant effect (*M* = 0.2°, *SE* = 0.6; one-sided *t* test against zero), while subjective PS showed the strongest adaptation (*M* = −2.6°, *SE* = 0.6, *t* = −4.6, *p* ≤ .001). The subjective AnP (averaged result of all seven targets, without pointing) measured by the verbal two alternative forced choice, that is, without pointing, showed a small but significant auditory shift opposite to the prismatic shift (*M* = −2.0°, *SE* = 0.7, *t* = −3.1, *p* = .002). The subjective AP (averaged result of all seven targets, with pointing) deviated significantly from the prismatic shift (*M* = −2.0°, *SE* = 0.6, *t* = −3.5, *p* = .001; Tables S7 and S8).

### Experiment 2: Auditory Localization During Adaptation in the Light

We measured the adaptation of felt eye (VS) and arm straight ahead position (PS), the subjective auditory shift (AnP and AP), as well as the baseline-corrected direct effects and aftereffects with right- and left-shifting prisms in the light, since in the light, aftereffects are generally smaller, but unconscious head rotation is larger and hence effects on auditory localization may be larger. Measuring the individual components of adaptation (visual versus head proprioceptive) should clarify whether the shift in acoustic localization is of genuinely acoustic nature.

#### Direct effect and aftereffect in the light

As in Experiment 1 (direct effect, aftereffect), a one-way ANOVA for repeated measurements was performed first, *F*(1, 52) = 334.9, *p* ≤ .001, then a two-sided *t* test for paired samples as post hoc analysis was calculated. The post hoc analysis indicated a significant difference between the direct effect and the aftereffect (*t* = 17.0, *p* ≤ .001; Table S10). Figure 5 shows the averaged and baseline-corrected direct effect (*M* = 9.5°, *SE* = 0.7, *t* = 13.4, *p* ≤ .001 one-sided *t* test against zero) measured with the room lights on. The results indicate a significant deviation in the direction of the prismatic shift. The mean direct effect amounts to 67% of the prismatic power (Tables S11 and S12).

Aftereffects in the light were significant (*M* = −4.9°, *SE* = 0.8, *t* = −6.1, *p* ≤ .001) deviating from straight ahead by 34% of the prismatic power (Figure 5; Tables S11 and S12).

#### Adaptive components in the light

Again, a one-way ANOVA for repeated measurements was performed first (results see adaptive components Experiment 1), and then two-sided *t* tests for paired samples as post hoc analysis were calculated. Both the subjective AP and AnP did not differ significantly from the subjective PS and the subjective VS, respectively, for both prisms (Tables S13 and S14).

The subjective VS in bright light deviated significantly in the direction of the prismatic shift (*M* = 1.4°, *SE* = 0.7, *t* = 1.8, *p* = .044; one-sided *t* test against zero; Figure 6). The subjective PS adapted significantly (*M* = −2.4°, *SE* = 0.6, *t* = −4.2, *p* = .001) opposite to the direction of the prismatic shift. The subjective AnP (two alternative forced choice, without pointing) showed a significant shift in the direction of the prismatic shift (*M* = 2.6°, *SE* = 1.2, *t* = 2.2, *p* = .025). The subjective AP (averaged result of all seven targets, with pointing) showed significant deviations opposite to the direction of the prismatic shift (*M* = −2.6°, *SE* = 0.6, *t* = −4.0, *p* = .001; Tables S15 and S16).

### Dark Condition Versus Light Condition

#### Direct effect and aftereffect

A two-way ANOVA for repeated measurements indicated neither for interaction prism direction by group, *F*(1,25) = 1.0, *p* = .327, nor for test by group, *F*(1,25) = 0.0, *p* = .860, any significant difference. Hence, the direct effect and the aftereffect in the dark did not differ significantly between the dark and light conditions for both right- and left-shifting prisms.

#### Adaptive components

A two-way ANOVA for repeated measurements indicated that the interaction prism direction by group, *F*(1, 25) = 1.0, *p* = .332, was not significant. However, the interaction posttest by group, *F*(3, 75) = 3.5, *p* = .020, showed significant differences. A post hoc analysis (two-sided unpaired *t* test) of the subjective AnP for left-shifting prisms (*t* = 3.1, *p* = .005) was significant while for right-shifting prisms (*t* = −1.9, *p* = .065), a trend existed. The subjective VS after adaptation failed to reach significance both for right-shifting prisms and for left-shifting prisms. The same was true for subjective PS and the subjective AP (Table S17).

### Experiment 3: Influence of Head Rotation on Auditory Localization

Rotation of the head of course changes the interaural differences both of arrival times and intensities. Head rotation therefore has to be taken into account when acoustic sources have to be localized. Since we attribute a large part of visual adaptation to head rotation which remains subconscious, we expect that the amount of unconscious head rotation that occurs during prism adaptation will influence the perceived position of acoustic sources. Here, we test whether the amount of unconscious head rotation corresponds quantitatively with the amount of acoustic adaptation. We compared the adaptive components (HO, PS, VS, AnP, and AP) as well as the results of the direct effect and the aftereffect in bright conditions.

#### Direct effect and aftereffect

**Figure 7.**
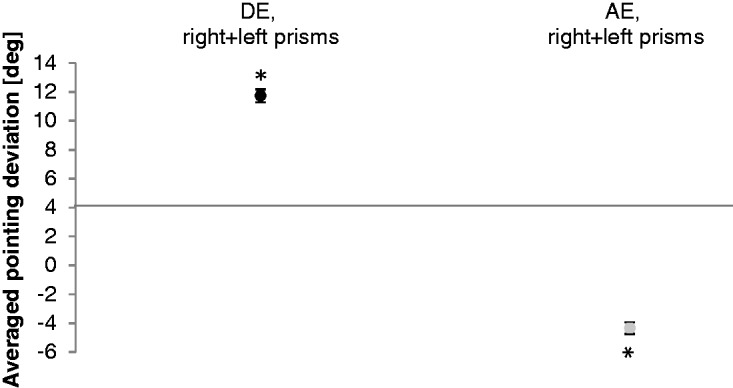
Direct effect (DE) and aftereffect (AE). Mean results of the right- and left-shifting prisms. Black dots show the averaged direct effect and gray dots the averaged aftereffect in the bright conditions. Error bars denote SEs. Asterisks indicate significant differences (*p* < .05).

Figure 7 illustrates the direct effects and the aftereffects measured in light conditions. A two-way analysis for repeated measurements with prism direction (right, left) and test (direct effect, aftereffect) as within-subject factors was calculated. For statistical analysis, results for left-shifting prisms were inverted. The results for the main effect prism direction, *F*(1, 10) = 0.3, *p* = .580, indicated no significant difference between both prism orientations. Therefore, the results of both prism orientations were combined (mean of right and left prisms).

A one-way ANOVA for repeated measurements with test (direct effect, aftereffect) as within-subject factor was calculated. The main effect test, *F*(1, 21) = 1156.0, *p* ≤ .001, showed a significant difference between the components. A post hoc analysis by two-sided *t* tests for paired samples yielded in a significant difference between the direct effect and the aftereffect (*t* = 34.0, *p* ≤ .001; Table S19).

The baseline-corrected and averaged result of the direct effect (*M* = 11.7°, *SE* = 0.5, *t* = 26.1, *p* ≤ .001; one-sided *t* test against zero; see Figure 7) showed a significant initial deviation from the central target. The direct effect amounted to 83% of the prismatic power (Tables S20 and S21).

Aftereffects under bright conditions were significant (*M* = −4.4°, *SE* = 0.4, *t* = −10.8, *p* ≤ .001; one-sided *t* test against zero) in the direction opposite to the prismatic shift. The aftereffect in the bright room was only 31% of prismatic power (Figure 7; Tables S20 and S21).

### Adaptive Components

**Figure 8.**
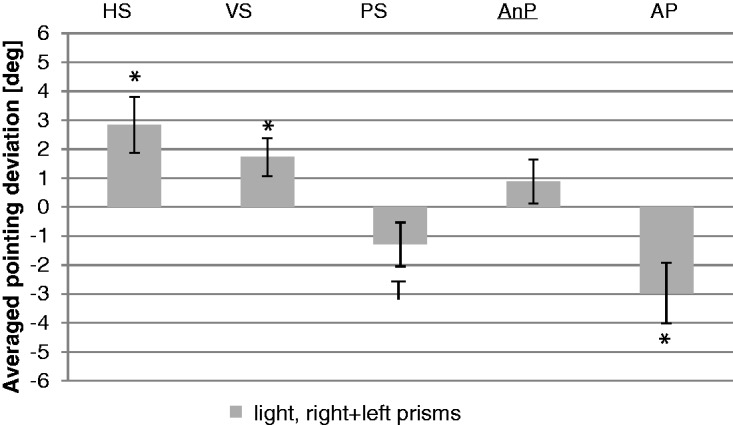
Adaptive components after prism adaptation (first posttest). Mean results of the right- and left-shifting prisms. Bars indicate the averaged results for the adaptive components (head straight ahead [HS], visual straight ahead [VS], proprioceptive straight ahead [PS], nonproprioceptive auditory straight ahead [AnP], and proprioceptive auditory straight ahead [AP]). Error bars indicate SEs. Asterisks indicate significant differences (*p* < .05) and *T*s indicate trends (*p* < .1).

Figure 8 shows subjective head (HS), eye (VS), and arm straight ahead position (PS) as well as the subjective auditory shifts (AnP and AP) measured in the light. A two-way analysis for repeated measurements with prism direction (right, left) and posttest (HS, VS, PS, AnP, and AP) as within-subject factors (for statistical analysis, results for left-shifting prisms were inverted), the results for the main effect prism direction, *F*(1, 8) = 0.6, *p* = .449, indicated no significant difference between the two prism orientations. Therefore, we combined the results of both prism orientations (mean of right and left prisms).

After calculating a one-way ANOVA for repeated measurements with posttest (HS, VS, PS, AnP, and AP) as within-subject factor, the main effect, *F*(4, 68) = 5.7, *p* = .001, showed a significant difference between the different components of adaptation. Two-sided *t* tests for paired samples as post hoc analysis showed that the subjective AP did not differ significantly from the subjective PS, and neither differed the subjective AnP from the subjective VS (Tables S22 and S23).

The subjective head straight ahead position (*M* = 2.8°, *SE* = 1.0; *t* = 2.9; *p* = .004; one-sided t-test against zero) significantly differed from objective straight ahead in the direction of the prismatic shift. The same was true for the subjective VS (*M* = 1.7°, *SE* = 0.7; *t* = 2.6; *p* = .008). The subjective AP (*M* = −3.0°, *SE* = 1.0; *t* = −2.8; *p* = .005; averaged results of all seven targets, without pointing) resulted in a significant effect opposite to the prismatic shift. However, the subjective PS (*M* = −1.3°, *SE* = 0.8, *t* = −1.7, *p* = .052) indicated a trend in the same direction. The subjective AnP (*M* = 0.9°, *SE* = 0.8; averaged results of all seven targets, with pointing) did not differ significantly from zero (Figure 8; Tables S24 and S25).

**Figure 9.**
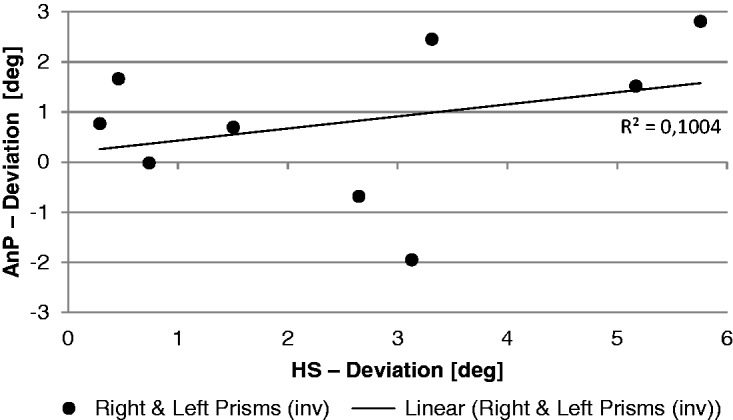
Comparison of the coherence between the head straight ahead (HS) and the nonproprioceptive auditory straight ahead (AnP) after prism adaptation (first posttest). Mean results of the right- and left-shifting prisms.

The correlation between the shift in the head straight ahead and the AnP fitted by a linear function *f*(*x*) = *ax* + *b* failed to reach significance (two-sided *t* test; Figure 9).

## Discussion

We investigated whether prism adaptation, that is, a realignment between the visual world and proprioception of the arm/hand system under the influence of prisms, has effects on the acoustic localization of objects. In a seminal article, Harris (1965) listed four basic ways how the human nervous system could adapt to the shift induced by prisms. Among these were a conscious cognitive correction of one’s hand movements, altered arm-proprioception, altered motor programs, and altered space perception. For both the motor programs and the space perception, he identified two possibilities: a purely visual type of adaptation or else a shift in the representation of extrapersonal space that would include the acoustic domain. The visual and acoustic domains certainly are closely connected to each other as we know not only from looking at a ventriloquist. As outlined in the Introduction section, in order to interact with objects, we have to localize them in space. This localization is based on both visual and acoustic information, and we generally form an internal space representation based on both of these types of information (McLaughlin & Bower, 1965; McLaughlin & Rifkin, 1965; Hay & Pick, 1966).

Based on this fact, one could expect that a change in visually perceived spatial positions should have an influence on the perceived spatial positions of acoustic sources, and that is in fact what a number of studies found (Callan et al., 2015; Chen & Spence, 2017; Radeau & Bertelson, 1974; Bosen et al., 2017), while others failed to find this generalization between modalities (Harris, 1963; Hein & Held, 1960; Pick et al., 1963).

To discriminate between these two basic possibilities—change of only visual senso-motor coupling versus visuo-acoustic-motor coupling—we performed three experiments. In the first two experiments, participants adapted to right-shifting or left-shifting prisms while sitting in the dark (Experiment 1) or else with room lights switched on (Experiment 2). We found larger direct effects of prisms in the dark than in the light, in line with earlier results (Pochopien & Fahle, 2015), and significant aftereffects after removing the prisms. Adaptation also produced a significant change in the felt position of the arm (in the direction opposite to the optical shift) and a change of similar size when participants had to indicate the position of the acoustic source by pointing in the corresponding direction. These two aftereffects, however, did not differ significantly. Hence, we conclude that the change in arm proprioception is sufficient to explain why participants mispointed when indicating the direction of the acoustic source. No change in the acoustic map is necessary.

This conclusion is further corroborated by the fact that we did not find a consistent shift of the perceived auditory direction when observers had to indicate, by pushing a button, whether a sound came from the right or from the left. In this task, arm position and its adaptation did not have an influence on the direction indicated. In the dark, this measurement does show a significant difference in direction opposite to the prismatic shift, that is, an aftereffect. But in the light condition, the direction of this “aftereffect” reverses direction. Hence, when the results of the nonproprioceptive auditory localization are combined, no (significant) indications for a shift of the auditory map result. We attribute this difference between the dark and bright conditions on different head positions after adaptation under the two different conditions.

From a previous study (Pochopien, Spang, Stemmler, & Fahle, 2017), we know that observers turn their head in the direction of the prismatic shift without being aware of this head rotation. In this way, they decrease the perceived displacement caused by the prisms, and their pointing movements deviate from target far less than to be expected from the power of the prisms. This immediate (compensation) effect is much larger under bright conditions than in the dark. Rotating the head subconsciously, with a significant shift in the direction of the subjective straight ahead position changes not only the subjective VS but also the subjective auditory straight ahead by the same amount.

It is reassuring that in Figure 6, the shift for the nonproprioceptive auditory shift was in the same direction as the visual shift for the light condition (and that the two do not differ significantly from each other). In the dark condition, VS was virtually unchanged (as in previous experiments: Pochopien & Fahle, 2015; Pochopien et al., 2017), so we did not expect any effect.

We decided to further investigate the influence of the head rotation in the third experiment. Again, we found significant direct effects and aftereffects. Unfortunately, head rotation after prism adaptation failed to reach significance for the right-shifting prisms but when both prism directions collapsed, head straight ahead deviated significantly from zero. Both for right- and left-shifting prisms, the AnP deviated from objective straight ahead in the same direction as the VS, though the variance was unfortunately very high. But the AnP deviated in the direction opposite to the AP, arguing against a change in the acoustic map. Combining the results for both prism orientations reveals significant adaptations of head rotation and subjective VS as well as a trend for PS, while not for AnP.

The significant effect on AP is in the order of arm PS. Hence, in spite of the high variance of results typical for experiments on prism adaptation, we can safely conclude that prism adaptation does not generalize to the acoustic map, but that any apparent generalization of adaptation from the visual to the acoustic domain can be explained completely by subconscious adaptations of head rotation and arm or hand proprioception.
